# Triggering Receptor Expressed on Myeloid Cells 2 Overexpression Inhibits Proinflammatory Cytokines in Lipopolysaccharide-Stimulated Microglia

**DOI:** 10.1155/2017/9340610

**Published:** 2017-10-18

**Authors:** Xiaobao Zhang, Fang Yan, Jizheng Cui, Yong Wu, Hengfei Luan, Miaomiao Yin, Zhibin Zhao, Jiying Feng, Jinwei Zhang

**Affiliations:** ^1^Department of Anesthesiology, The Affiliated Lianyungang Hospital of Xuzhou Medical University, 182 Tongguan Road, Lianyungang 222000, China; ^2^Department of Basic Medical Science, Kangda College, Nanjing Medical University, 88 Chunhui Road, Lianyungang 222000, China; ^3^Department of Anesthesiology, Affiliated Drum Tower Hospital of Nanjing University Medical School, 321 Zhongshan Road, Nanjing 210008, China

## Abstract

Microglia play an important role in mediating inflammatory processes in the central nervous system (CNS). Triggering receptor expressed on myeloid cells 2 (TREM2) is a microglia-specific receptor and could decrease neuropathology in Alzheimer's disease (AD). However, the detailed mechanism remains unclear. This study was designed to elucidate the effect of TREM2 on microglia. We showed that lipopolysaccharide (LPS) stimulation significantly increases proinflammatory cytokines and suppressed TREM2 in microglia. In addition, TREM2 overexpression inhibited LPS-induced microglia activation and elevated M2 phenotype of microglia. Together, our results demonstrate that TREM2 overexpression reduced LPS-induced proinflammatory cytokine release in microglia and increased M2 phenotype of microglia. These findings provide novel insights that the regulation of microglia polarization may be an approach for ameliorating microglia inflammation in neurodegenerative diseases.

## 1. Introduction

Neuroinflammation plays a pivotal role in the pathophysiology of neurocognitive disorders such as AD and Parkinson's disease (PD) [1]. Both epidemiological and genetic studies support a pivotal role of neuroinflammation in the pathophysiology of neurocognitive disorders [1, 2]. Lipopolysaccharide (LPS) is one of the most potent activators of systemic inflammation for stimulating proinflammatory cytokine release in experimental animals and humans [3]. Systemic inflammation could induce neuroinflammation and result in memory impairment and progressive neurodegeneration [4, 5].

Microglia are the resident immune cells in the brain and exert protective responses to inflammation in the CNS [6]. They play a critical role in neurodegenerative disease. Inhibition of the excessive microglial proinflammatory response may alleviate the symptom of neurodegenerative diseases [7, 8]. Microglia can be activated by stimulation such as LPS, exerting high expression of proinflammatory cytokines (i.e., TNF-*α*, IL-6, and IL-1*β*) [4].

Triggering receptor expressed on myeloid cells 2 (TREM2) is a cell-surface receptor involved in transport and phagocytosis. It is found in microglia, osteoclasts, and macrophages in vivo as well as macrophage cell lines in vitro [9, 10]. It couples with DAP12, an associated transmembrane adapter, to trigger cell activation for its signaling and biological functions [11, 12]. An in vivo study found that overexpressing TREM2 ameliorated neuronal and synaptic loss and alleviated cognitive impairments in P301S mice [13]. Moreover, an in vitro study showed that reduced microglial TREM2 expression impaired phagocytosis of apoptotic neurons [14]. However, it has been unclear whether TREM2 overexpression has salutary effects on the primary microglia. The primary aim of this study was to explore the role of TREM2 in LPS-induced proinflammatory mediator production in primary microglia.

## 2. Material and Methods

### 2.1. Primary Microglia Culture

Primary microglia were prepared as in previous study with a slight modification [15]. Briefly, the cerebellum of postnatal 1- to 3-day-old mice was dissected and the meninges were removed. After mashing with trypsin and centrifuging, the cerebral cortices were passed through 100 *μ*m and 40 *μ*m meth sequentially. Then, the mixed glial cells were plated and cultured in DMEM with 40 ng/mL MCSF (R&D Systems, Minneapolis, MN). Microglial cells were harvested by shaking the flask for 1 to 2 h after 10 to 12 days in culture. The microglia purity was detected by Iba-1(>90%). All media were replaced with serum-free DMEM before the experiment.

### 2.2. Lentiviral Vector Preparation and Microglia Transduction

The mouse TREM2 gene lentiviral vectors and control lentiviral vectors were constructed as described in a previous study [13]. Then, the TREM2 lentiviral vectors were transfected with packaging vectors into 293FT cells. After 48 h culture, the lentiviral particles in the supernatant were collected and concentrated. The lentiviral particle titers were determined by ELISA kit. Primary microglia were seeded at 5 × 10^5^ cells/well per mL into 12-well plates with incubation medium. After adding the lentiviral particle to the culture, the supernatant was replaced with DMEM containing 10% fetal calf serum after 2 h infection. The transduction efficiency was determined by polymerase chain reaction (PCR) and Western blot analysis. The microglia were pretreated with LPS (Sigma, St. Louis, MO, 100 ng/mL) and/or TREM2 lentivirus before incubation.

### 2.3. Real-Time Polymerase Chain Reaction

Total microglial RNA was extracted with RNAiso Plus and then converted to cDNA. The cDNA of microglia was used as a template for PCR (7300 PCR System). Then, the cDNA was amplified by PCR with primers of TNF-*α* (sense primer: 5′-AGC CCA CGT CGT AGC AAA CCA C-3′, antisense primer: 5′-AGG TAC AAC CCA TCG GCT GGC A-3′); IL-1*β* (sense primer: 5′-CCT GCA GCT GGA GAG TGT GGA T-3′, antisense primer: 5′-TGT GCT CTG CTT GTG AGG TGC T-3′); IL-6 (sense primer: 5′-CCT GCA GCT GGA GAG TGT GGA T-3′, antisense primer: 5′-TGT GCT CTG CTT GTG AGG TGC T-3′); Arg1 (sense primer: 5′-CTC CAA GCC AAA GTC CTT AGA G-3′, antisense primer: 5′-AGG AGC TGT CAT TAG GGA CAT C-3′); and IL-10 (sense primer: 5′-AGG CGC TGT CAT CGA TTT CTC-3′, antisense primer: 5′-TGC TCC ACT GCC TTG CTC TTA-3′) and *β*-actin (sense primer: 5′-TTG TAA CCA ACT GGG ACG ATA TGG-3′, antisense primer: 5′-GAT CTT GAT CTT CAT GGT GCT AG-3′). *β*-Actin was used as an internal control to evaluate the expression of inflammatory cytokines.

### 2.4. Western Blot Analysis

Microglial proteins were extracted using RIPA buffer (Sigma). The concentration of protein in the lysate supernatant fluid was measured by BCA protein assay. The gels were transferred to PVDF membranes after electrophoresis. Membranes were blocked with 5% bovine serum albumin and then incubated overnight with rabbit anti-TREM2 antibody (1 : 100; Santa Cruz) and anti-*β*-actin antibody (1 : 1000; Sigma). Horseradish peroxidase conjugated secondary antibodies (1 : 10,000; Jackson ImmunoResearch) were used to detect immunoreactivity. The density of the protein band was detected by image analysis system (Image-Pro Plus version 6.0).

### 2.5. Statistical Analysis

The data were analyzed using one-sample *t*-test or one-way ANOVA with Graphpad Prism 5 software. The Newman-Keuls multiple comparison test was used for post hoc analysis. Data were represented as mean ± SEM and *P* < 0.05 was considered statistically significant.

## 3. Results

### 3.1. Effects on the Expression of TNF-*α*, IL-6, IL-1*β*, IL-10, and Arg1 Induced by LPS

**Figure 1:**
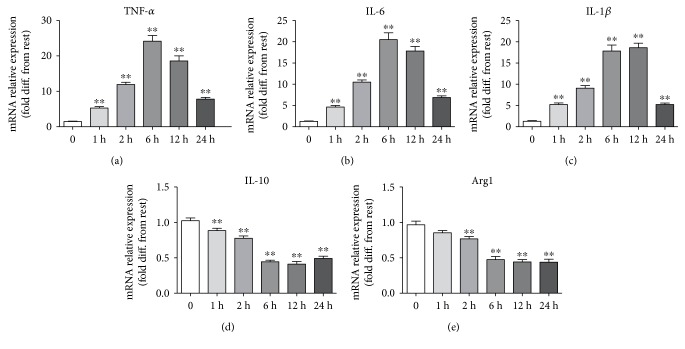
Effect of 100 ng/mL LPS on TNF-*α*, IL-6, IL-1*β*, IL-10, and Arg1 gene expression in primary microglia (^∗∗^*P* < 0.01 versus control group, each data represents the mean ± SEM of at least three separate experiments).

Our previous results indicated that 100 ng/mL could induce increase in inflammatory response in microglia and do not affect the viability of microglia. Therefore, 100 ng/mL was used in this study. After stimulation with LPS, microglial cells produced significant increase of TNF-*α*, IL-6, and IL-1*β* in the media in a time-dependent manner before 12 hours and decreased in 24 hours (Figures 1(a), 1(b), and 1(c)).

Compared with those of the control group, the levels of TNF-*α*, IL-6, and IL-1*β* in the media were increased in microglia after being treated with 100 ng/mL LPS. Moreover, the levels of IL-10 and Arg1 in the media were obviously decreased (Figures 1(d) and 1(e)).

### 3.2. Effect of LPS on TREM2 Expression in Microglia

**Figure 2:**
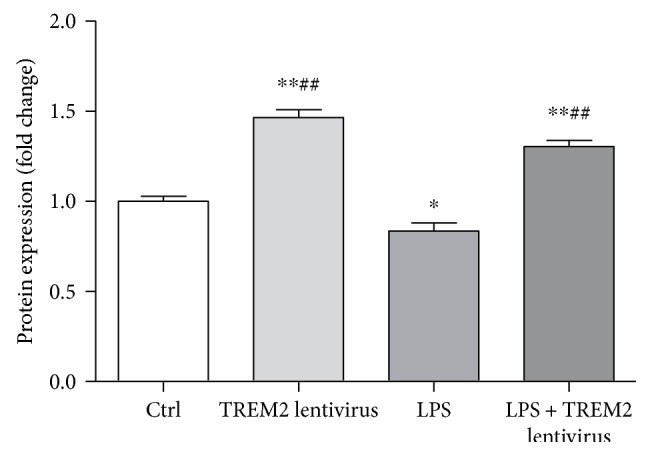
LPS decreases TREM2 expression and TREM2 lentivirus increases TREM2 expression in microglia. Primary microglia were treated with LPS (100 ng/mL) for 12 h. Cell lysates were analyzed by Western blotting. Microglia treated with LPS showed decrease in TREM2 levels. Overexpression of TREM2 showed a marked increase in TREM2 levels compared with LPS alone (^∗^*P* < 0.05, ^∗∗^*P* < 0.01 versus control group; ^##^*P* < 0.01 versus LPS group).

To evaluate the influence of LPS and TREM2 lentivirus on TREM2 expression in microglia, the microglia were pretreated with LPS and/or TREM2 lentivirus before incubation. Our results indicated that LPS inhibited microglial TREM2 expression and TREM2 lentivirus significantly increase TREM2 expression (Figure 2).

### 3.3. TREM2 Overexpression Inhibited LPS-Induced Microglia Activation and Elevated M2 Phenotype of Microglia

**Figure 3:**
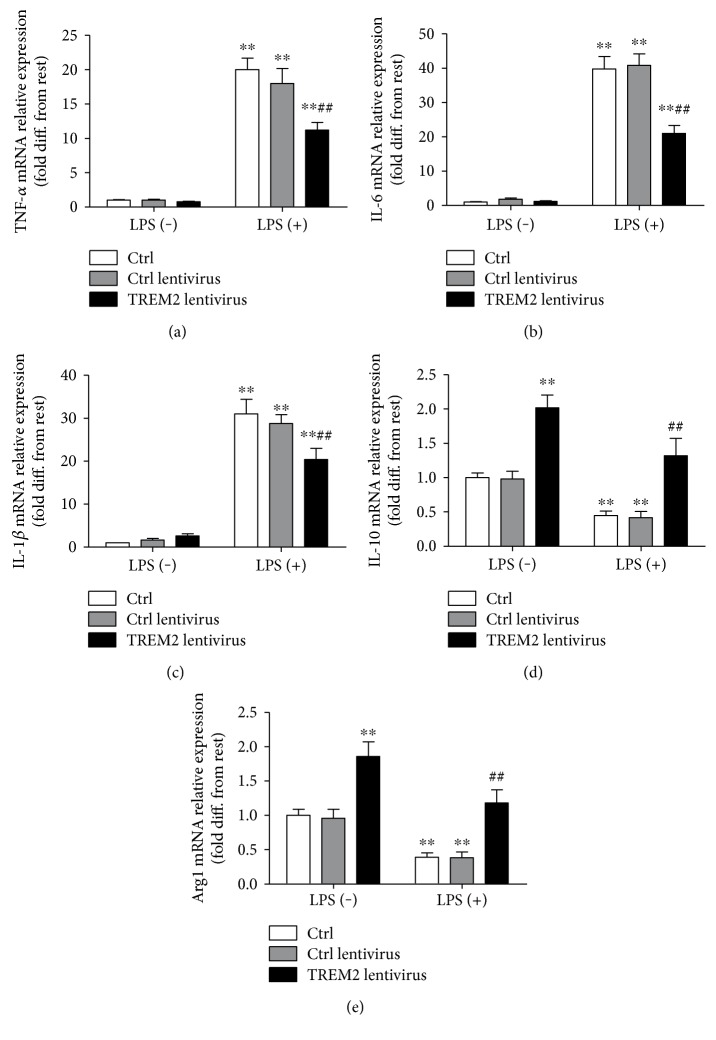
Overexpression of TREM2 in primary microglia attenuated LPS-induced elevation of TNF-*α*, IL-6, and IL-1*β* (a, b, c) and increased IL-10 and Arg1 level with/without LPS (d, e). Microglia were treated with TREM2 lentivirus before LPS stimulation. The level of M1 and M2 phenotype of microglia was detected by RT-PCR (^∗∗^*P* < 0.01 versus control group, ^##^*P* < 0.01 versus LPS treatment group).

To elucidate the effect of TREM2 in microglial polarization, we use lentivirus to overexpress TREM2. Our results showed that in response to LPS, overexpression of microglial TREM2 restrains the production of proinflammatory cytokines (TNF-*α*, IL-6, and IL-1*β*) (Figures 3(a), 3(b), and 3(c)). However, overexpression of TREM2 in microglia increased the M2 polarization of microglia (Arg1 and IL-10) (Figures 3(d) and 3(e)).

## 4. Discussion

The present study demonstrates that LPS could induce microglial activation, and overexpression of TREM2 could suppress LPS-induced microglial activation.

Microglial cells are innate immune mediators in CNS and could release proinflammatory mediators [16]. Generally, activated microglia are referred to M1-like and M2-like [17]. The M1 phenotype is featured by enhanced proinflammatory activity, whereas M2 phenotype of microglia exerts phagocytic activity as well as neuroprotective effect [18].

Studies showed that systemic LPS is connected with memory impairment [19, 20], chronic neuroinflammation, and progressive neurodegeneration [4]. Hypoxia, acidosis, and LPS administration favored the primary microglia to the proinflammatory M1 phenotype, whereas 17-beta-estradiol (E2) and progesterone shifted the microglia to the neuroprotective M2 phenotype [21]. In PD and AD model rats, microglia mainly express M1-like cytokines than M2 [22–24]. Mesenchymal stem cells (MSCs) exert a neuroprotective effect by controlling microglia M2 polarization [25]. However, in some other diseases, the cytokines and antigens expressed by activated microglia cut across the M1 and M2 categories [26, 27].

TREM2 is mainly expressed on microglia and functions in the human brain including hippocampus as a hub gene. TREM2 plays a key role in the microglial phagocytosis and migration [28, 29]. The level of TREM2 is upregulated in the cells surrounding A*β* plaques in the CNS of AD models mice [30]. AD and other neurodegeneration diseases are most probably due to reduced shedding or dysfunction of TREM2 [11, 31]. The role of TREM2 in the pathogenesis of neurodegeneration disease remains unclear.

In addition to TREM2, several other receptors of TREM family, such as TREM1, are also involved in regulating phenotype of microglia as well as microglial cytokine release. While TREM1 activation promotes the secretion of proinflammatory cytokine reduction, TREM2 activation instead suppresses LPS-induced proinflammatory effects [32, 33].

To explore whether TREM2 affected microglial polarization, local lentivirus was injected into media of microglia to overexpress TREM2. Lipopolysaccharides are considered to be a potent stimulator-induced TREM2/Dap12 gene expression and significantly exacerbated proinflammatory responses [34]. Our results revealed that TREM2 overexpression induced M2 phenotype and inhibited the inflammatory response of microglia. This observation was consistent with a previous study which showed that overexpression of TREM2 protected neuron injury and prevents synaptic loss in the brain [35].

Moreover, several studies also revealed that overexpression of either full-length TREM2 or C-terminal fragment of TREM2 reduced LPS-induced inflammatory responses [33], and TREM2 deficiency disrupts the microglia barrier formation and regulates amyloid insulation and compaction [36]. Trem2−/− microglia showed reduced transcripts indicative of phagocytosis and lipid catabolism [37]. During early to middle stage of AD, TREM2 showed neuroprotective effect [38], whereas in aging mice, TREM2 overexpression fails to provide neuroprotection effect because of microglial A*β* phagocytosis deficit at the late stage of disease progression.

Our study also suffered several limitations; we demonstrated the effect of TREM2 on cytokines with overexpression; however, to knockdown TREM2 and assess its effect on microglial phenotype, proinflammatory cytokine release is needed to elucidate the effect of TREM2 on microglial activation. In addition, the signaling pathway of TREM2 remains unknown and further studies are required to illustrate the underlying mechanisms of TREM2 which modulate polarization.

In conclusion, our findings demonstrate that TREM2 overexpression reduced LPS-induced proinflammatory cytokine release in microglia and increased M2 phenotype of microglia. These findings provide novel insights that the regulation of microglia polarization may be an approach for ameliorating microglia inflammation in neurodegenerative diseases.

## References

[1] Heneka M. T., Carson M. J., El Khoury J. (2015). Neuroinflammation in Alzheimer’s disease. *Lancet Neurology*.

[2] Calvello R., Cianciulli A., Nicolardi G. (2017). Vitamin D treatment attenuates neuroinflammation and dopaminergic neurodegeneration in an animal model of Parkinson’s disease, shifting M1 to M2 microglia responses. *Journal of Neuroimmune Pharmacology*.

[3] Li Q., Dong D. D., Huang Q. P. (2017). The anti-inflammatory effect of *Sonchus oleraceus* aqueous extract on lipopolysaccharide stimulated RAW 264.7 cells and mice. *Pharmaceutical Biology*.

[4] Qin L., Wu X., Block M. L. (2007). Systemic LPS causes chronic neuroinflammation and progressive neurodegeneration. *Glia*.

[5] Giuliani D., Ottani A., Neri L. (2017). Multiple beneficial effects of melanocortin MC4 receptor agonists in experimental neurodegenerative disorders: therapeutic perspectives. *Progress in Neurobiology*.

[6] Ransohoff R. M., Brown M. A. (2012). Innate immunity in the central nervous system. *The Journal of Clinical Investigation*.

[7] Hirsch E. C., Vyas S., Hunot S. (2012). Neuroinflammation in Parkinson’s disease. *Parkinsonism & Related Disorders*.

[8] Maccioni R. B., Rojo L. E., Fernandez J. A., Kuljis R. O. (2009). The role of neuroimmunomodulation in Alzheimer’s disease. *Annals of the New York Academy of Sciences*.

[9] Colonna M., Wang Y. (2016). TREM2 variants: new keys to decipher Alzheimer disease pathogenesis. *Nature Reviews Neuroscience*.

[10] Ulrich J. D., Holtzman D. M. (2016). TREM2 function in Alzheimer’s disease and neurodegeneration. *ACS Chemical Neuroscience*.

[11] Walter J. (2016). The triggering receptor expressed on myeloid cells 2: a molecular link of neuroinflammation and neurodegenerative diseases. *The Journal of Biological Chemistry*.

[12] Frank S., Burbach G. J., Bonin M. (2008). TREM2 is upregulated in amyloid plaque-associated microglia in aged APP23 transgenic mice. *Glia*.

[13] Jiang T., Zhang Y. D., Chen Q. (2016). TREM2 modifies microglial phenotype and provides neuroprotection in P301S tau transgenic mice. *Neuropharmacology*.

[14] Takahashi K., Rochford C. D., Neumann H. (2005). Clearance of apoptotic neurons without inflammation by microglial triggering receptor expressed on myeloid cells-2. *The Journal of Experimental Medicine*.

[15] Zhang X., Wang J., Qian W. (2015). Dexmedetomidine inhibits inducible nitric oxide synthase in lipopolysaccharide-stimulated microglia by suppression of extracellular signal-regulated kinase. *Neurological Research*.

[16] Kettenmann H., Hanisch U. K., Noda M., Verkhratsky A. (2011). Physiology of microglia. *Physiological Reviews*.

[17] Mosser D. M., Edwards J. P. (2008). Exploring the full spectrum of macrophage activation. *Nature Reviews Immunology*.

[18] Zhang Q., Lu Y., Bian H., Guo L., Zhu H. (2017). Activation of the α7 nicotinic receptor promotes lipopolysaccharide-induced conversion of M1 microglia to M2. *American Journal of Translational Research*.

[19] Lee Y. J., Choi D. Y., Choi I. S. (2012). Inhibitory effect of 4-*O*-methylhonokiol on lipopolysaccharide-induced neuroinflammation, amyloidogenesis and memory impairment via inhibition of nuclear factor-kappaB *in vitro* and *in vivo* models. *Journal of Neuroinflammation*.

[20] Hsing C. H., Hung S. K., Chen Y. C. (2015). Histone deacetylase inhibitor trichostatin A ameliorated endotoxin-induced neuroinflammation and cognitive dysfunction. *Mediators of Inflammation*.

[21] Habib P., Slowik A., Zendedel A., Johann S., Dang J., Beyer C. (2014). Regulation of hypoxia-induced inflammatory responses and M1-M2 phenotype switch of primary rat microglia by sex steroids. *Journal of Molecular Neuroscience*.

[22] Zhu D., Yang N., Liu Y. Y., Zheng J., Ji C., Zuo P. P. (2016). M2 macrophage transplantation ameliorates cognitive dysfunction in amyloid-β-treated rats through regulation of microglial polarization. *Journal of Alzheimer's Disease*.

[23] Blandini F. (2013). Neural and immune mechanisms in the pathogenesis of Parkinson’s disease. *Journal of Neuroimmune Pharmacology*.

[24] Wang S., Jing H., Yang H. (2015). Tanshinone I selectively suppresses pro-inflammatory genes expression in activated microglia and prevents nigrostriatal dopaminergic neurodegeneration in a mouse model of Parkinson’s disease. *Journal of Ethnopharmacology*.

[25] Park H. J., SH O., Kim H. N., Jung Y. J., Lee P. H. (2016). Mesenchymal stem cells enhance α-synuclein clearance via M2 microglia polarization in experimental and human parkinsonian disorder. *Acta Neuropathologica*.

[26] Colton C. A., Mott R. T., Sharpe H., Xu Q., Van Nostrand W. E., Vitek M. P. (2006). Expression profiles for macrophage alternative activation genes in AD and in mouse models of AD. *Journal of Neuroinflammation*.

[27] Perry V. H. (2010). Contribution of systemic inflammation to chronic neurodegeneration. *Acta Neuropathologica*.

[28] Colonna M. (2003). TREMs in the immune system and beyond. *Nature Reviews Immunology*.

[29] Forabosco P., Ramasamy A., Trabzuni D. (2013). Insights into TREM2 biology by network analysis of human brain gene expression data. *Neurobiology of Aging*.

[30] Matarin M., Salih D. A., Yasvoina M. (2015). A genome-wide gene-expression analysis and database in transgenic mice during development of amyloid or tau pathology. *Cell Reports*.

[31] Villegas-Llerena C., Phillips A., Garcia-Reitboeck P., Hardy J., Pocock J. M. (2016). Microglial genes regulating neuroinflammation in the progression of Alzheimer’s disease. *Current Opinion in Neurobiology*.

[32] Takahashi K., Prinz M., Stagi M., Chechneva O., Neumann H. (2007). TREM2-transduced myeloid precursors mediate nervous tissue debris clearance and facilitate recovery in an animal model of multiple sclerosis. *PLoS Medicine*.

[33] Zhong L., Chen X. F., Zhang Z. L. (2015). DAP12 stabilizes the C-terminal fragment of the triggering receptor expressed on myeloid cells-2 (TREM2) and protects against LPS-induced pro-inflammatory response. *The Journal of Biological Chemistry*.

[34] Pride J. R. (1989). Good leadership skills make top less lonely. Interview by Hugh Doherty. *Dentistry Today*.

[35] Jiang T., Tan L., Zhu X. C. (2014). Upregulation of TREM2 ameliorates neuropathology and rescues spatial cognitive impairment in a transgenic mouse model of Alzheimer’s disease. *Neuropsychopharmacology*.

[36] Yuan P., Condello C., Keene C. D. (2016). TREM2 haplodeficiency in mice and humans impairs the microglia barrier function leading to decreased amyloid compaction and severe axonal dystrophy. *Neuron*.

[37] Poliani P. L., Wang Y., Fontana E. (2015). TREM2 sustains microglial expansion during aging and response to demyelination. *The Journal of Clinical Investigation*.

[38] Jiang T., Wan Y., Zhang Y. D. (2017). TREM2 overexpression has no improvement on neuropathology and cognitive impairment in aging APPswe/PS1dE9 mice. *Molecular Neurobiology*.

